# Balancing Selectivity and Cross-Sensitivity in Smart Tongue Sensing—Multimode, Multiplexed Optical Sensor Arrays for Dietary Supplement Classification

**DOI:** 10.3390/s26196312

**Published:** 2026-10-06

**Authors:** Aleksandra Kossakowska, Emilia Stelmach, Anna Kisiel, Krzysztof Maksymiuk, Agata Michalska, Patrycja Ciosek-Skibińska

**Affiliations:** 1 Chair of Medical Biotechnology, Faculty of Chemistry, Warsaw University of Technology, Noakowskiego 3, 00-664 Warsaw, Poland; 2 Faculty of Chemistry, University of Warsaw, Pasteura 1, 02-093 Warsaw, Poland

**Keywords:** microparticles, nanoparticles, nano-optodes, smart tongue, machine learning, differential sensing, dietary supplements

## Abstract

Achieving both universality and robustness in smart sensing remains a central yet unresolved challenge. Herein, we move beyond the conventional dichotomy of selective versus cross-sensitive sensors by demonstrating their synergistic integration within differential sensing architectures. Optical micro- and nanoparticle-based sensors—both selective (UW) and cross-sensitive (WUT)—were rationally combined to construct information-rich sensing arrays. Performance enhancement was explored considering three complementary strategies: the reduction of inter-sensor redundancy, the exploitation of full spectral fingerprints and multimode optical readout. Across independent datasets, log F analysis uncovered pronounced context-dependent chemosensitive particle behavior, enabling the identification of task-specific leading nanostructures while confirming metric robustness. Full-spectrum chemometric modeling delivered consistently high classification accuracy without overfitting, where SVM-DA excelled in resolving complex multiclass problems. Collectively, this work establishes a generalizable framework for engineering adaptive, high-dimensional sensing platforms, advancing the design of next-generation smart analytical systems, including smart noses and smart tongues.

## 1. Introduction

Traditionally, the field of chemical analysis has prioritized selective or specific sensors. These sensors are designed to interact with a single target analyte with high specificity, which provides clear and precise results in controlled laboratory settings [1,2]. However, this high level of selectivity can become a limitation when dealing with complex real-world matrices, such as clinical or environmental samples, where various interfering substances are present. In response to these limitations, a new paradigm has emerged that focuses on cross-sensitive sensors and differential detection, also known as “smart sensing systems” [3,4,5,6,7,8,9,10,11,12]. Instead of seeking one specific sensor for one analyte, this approach utilizes multifield sensor systems that generate a “fingerprint” of the sample. By analyzing the patterns of response from a diverse array of sensors, these systems can simultaneously detect multiple (bio)analytes within a complex mixture [13,14,15,16]. This “fingerprint sensing” significantly expands the potential for advanced analysis in diverse fields, ranging from medical diagnostics to environmental monitoring.

A great challenge in developing these systems is selecting the correct combination of sensors to ensure universality and flexibility. A sensor that performs exceptionally well in one specific scenario may limit the system’s effectiveness when applied to a different type of sample set. To address this, various strategies can be considered.

1. Reducing correlation—it is essential to choose sensors that provide distinct, low-correlation responses within the matrix; increasing the pool of available sensors (e.g., by considering also selective sensors) can help to reduce information redundancy and improve the overall quality of the data [17,18].
2. Multiplexing responses—this involves extracting multiple streams of information from a single sensing element (e.g., full spectrum instead of pointwise signal) [4,19,20,21,22]; this information-rich method, often referred to as a “virtual sensor array”, involves the use of multidimensional responses, thereby significantly improving the system’s ability to classify complex samples.
3. Multimode optical signals—using different reading modes, such as measuring both absorption and fluorescence from each sensor element (in the case of fluorescence, the fluorescence emission across various excitation wavelengths can additionally be considered) [10,23,24].

**Figure 1:**
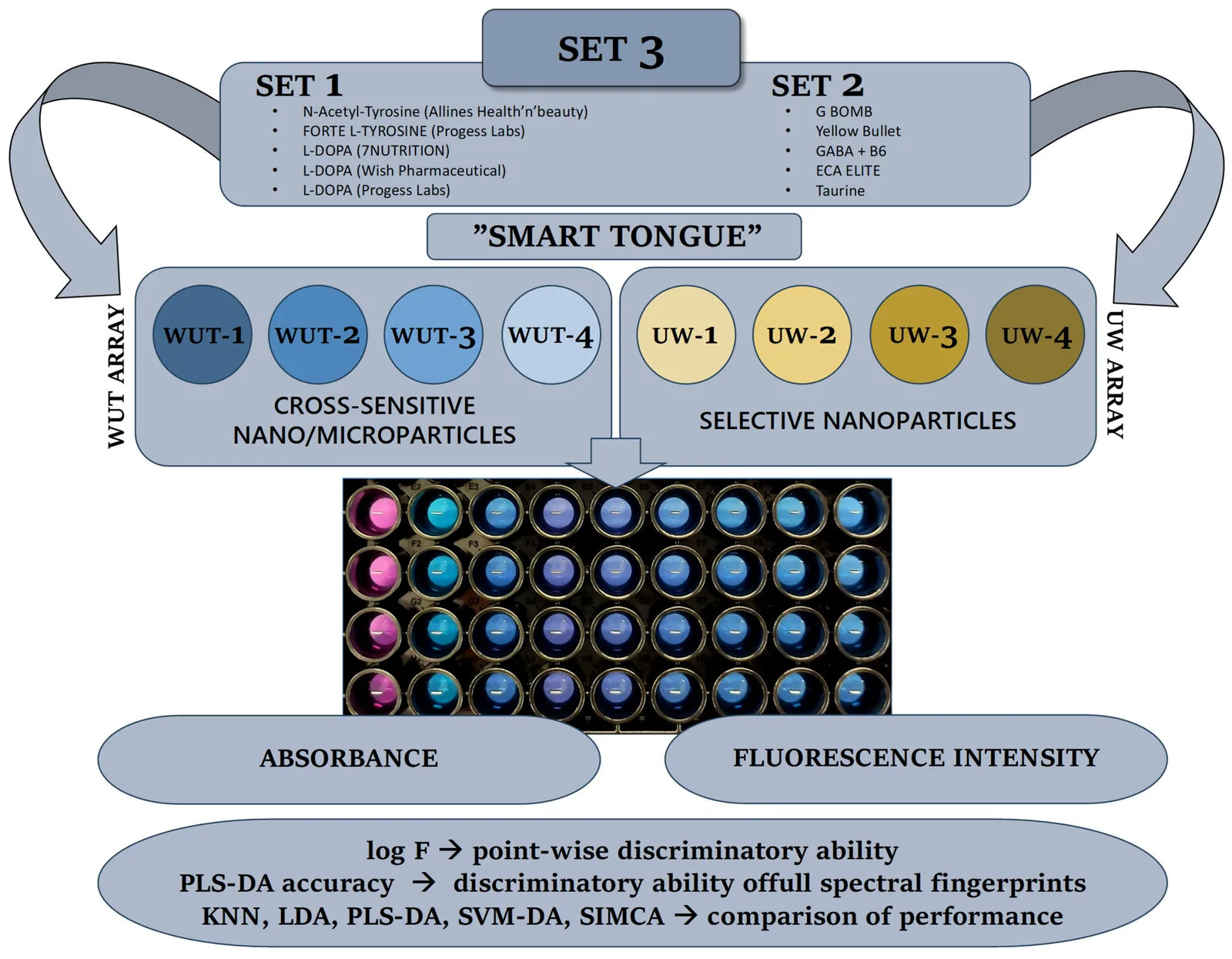
Scheme of the experimental workflow: micro- and nanoparticles served as detection units within a “smart tongue” designed for studies with three supplement sets (Table 1 and Table 2, respectively). For both single particles and the complete smart tongue, UV–Vis absorbance and fluorescence intensity measurements were performed. Point values, corresponding to the absorbance at the peak maximum and the fluorescence intensity at the emission maximum, were used to calculate log F, whereas the complete spectral fingerprints provided the basis for evaluating the accuracy of chemometric models.

This work aims to investigate and compare the performance of cross-sensitive and selective (WUT and UW type, respectively; Table 1, Figure 1) micro- and nanoparticles to determine whether the latter provide satisfactory discriminatory abilities for smart sensing strategies (the cross-sensitive particles were developed at Warsaw University of Technology—WUT—and the selective particles at the University of Warsaw—UW). Furthermore, this approach may help to reduce correlations among sensor responses while simultaneously providing more comprehensive information within smart sensing systems, when both sensor types, cross-sensitive and selective, are combined in the array. In addition, this study examines whether response multiplexing—by exploiting full spectral information—enhances performance to a similar extent for both selective and cross-sensitive sensors. Finally, the discriminatory abilities of the studied cross-sensitive and selective optical micro- and nanoparticles are compared across various measurement modes (spectrophotometric and spectrofluorimetric) and data analysis methods.

## 2. Experimental Section

### 2.1. Materials and Tested Dietary Supplement Samples

The compositions of the micro- and nanoparticles used in this study, together with their basic optical characteristics, are summarized in Table 1. The surfactants Pluronic F-127, Triton X-100 and poly(vinyl alcohol) PVA were supplied by Sigma-Merck (Poznań, Poland). Indium(III) 2,3,7,8,12,13,17,18-(octaethyl)porphyrin chloride (In(OEP)Cl) was supplied by PorphyChem (390 Rue Charles Freycinet, 21600 Longvic, France). The plasticizers 2-nitrophenyl octyl ether (o-NPOE) and bis(2-Ethylhexyl) sebacate (DOS); the lipophilic salts tridodecylmethylammonium chloride (TDMAC), potassium tetrakis3,5-bis(trifluoromethyl)phenyl borate (KTFPB) and sodium tetraphenylborate (NaTPB); and the chromoionophores Chromoionophore I (9-(diethylamino)-5-(octadecanoylimino)-5H-benzo[a]phenoxazine, ETH 5294), Chromoionophore II (9-dimethylamino-5-[4-(16-butyl-2,14-dioxo-3,15-dioxaeicosyl)phenylimino]benzo[a] phenoxazine, ETH 2439) and Chromoionophore XI (fluorescein octadecyl ester, ETH 7061) were obtained from Fluka (Selectophore, Warsaw, Poland). Rhodamine B octadecyl ester perchlorate (RBOE); poly [3-(6-carboxyhexyl)thiophene-2,5-diyl] (PHT-COOH), regioregular, MW 50–60 kDa; poly(3-octylthiophene-2,5-diyl) (POT); and calcium ionophore I (ETH1001) were supplied by Sigma-Merck (Poznań, Poland). Milli-Q water was used for the preparation of all aqueous solutions. Tetrahydrofuran (Fluka, Warsaw, Poland) was used as a solvent for the microparticle components. All chemicals were used as received.

The ten dietary supplements studied in this work, including their classification into SET 1 and SET 2, are summarized in Table 2. They are commercially available, produced by various manufacturers and can be purchased from various sources in Poland (supplement shops, pharmacies). The dietary supplements were stored in their original packaging at room temperature and were freshly prepared prior to the measurements. The chemical components used for the preparation of the WUT and UW micro-/nanoparticles were stored according to the manufacturers’ recommendations, either in a laboratory cabinet at room temperature or in a refrigerator, as applicable.

### 2.2. Preparation and Measurements of NPs/MPs

Eight types of micro-/nanoparticles were prepared from the components listed in Table 1. WUT particles (WUT-1—lipophilic anion-sensitive, WUT-2—lipophilic cation-sensitive, WUT-3—amine-sensitive, WUT-4—anion cross-sensitive with preferential affinity toward halides and selected organic anions with carboxyl groups) contained components such as plasticizers, surfactants or ion exchangers, while a metalloporphyrin served as both a receptor and optical transducer. These particles were prepared analogously to a protocol already used in our previous works [4,25,26]. The preparation involves dissolving all selected components in 1.5 mLof THF to obtain a homogeneous solution. To thoroughly dissolve the mixture, each vial was placed in an ultrasonic bath (Sonic-0.5, 80 W, 40 kHz, POLSONIC Palczyński Sp. J., Warsaw, Poland). The next step was to pipette 0.5 mL aliquots into 4.5 mL of deionized water on a vortex mixer. The final step involves passing air through the solution using a peristaltic pump (Gilson, Minipuls3, Warsaw, Poland). This process takes an hour, resulting in a clear suspension of micro-/nanoparticles ready for further testing.

For UW-1 Ca^2+^-sensitive nanostructure fabrication, 5 mL of a solution of aqueous 1% (*w*/*w*) PVA (31 kDa) was placed on a stirring plate (240 rpm) and 1.25 mL of PHT-COOH solution in THF (saturated, 0.80 mg/mL) was added, followed by 750 μL of THF solution containing 6.93 mg of ETH1001 and 2.40 mg of NaTFPB. The mixture was left on the stirring plate in an open vial for 30 min, resulting in Ca-PHT-COOH nanostructures [27]. In the case of UW-2 pH-sensitive nanostructures, 5 mL of a solution of aqueous 1% (*w*/*w*) PVA (31 kDa) was placed on a stirring plate (240 rpm) and 1.25 mL of PHT-COOH solution in THF (saturated, 0.80 mg/mL) was added. The mixture was left on the stirring plate in an open vial for 30 min, resulting in pH-PHT-COOH nanostructures [27]. For UW-3 redox-sensitive nanostructure preparation, 5 mL of a solution of aqueous 1% (*w*/*w*) PVA (31 kDa) was placed on a stirring plate (600 rpm). Then, 500 μL of POT solution in THF (10 mg/mL) was added. The mixture was left on the stirring plate in an open vial. Next, 1 mL of the obtained POT nanoparticle suspension was placed on the stirring plate (600 rpm) and 200 μL of THF solution containing 1 mg of NaTFPB and 15 mg of DOS in 1 mL was added. The mixture was left on the stirring plate in an open vial for 30 min [28]. In the case of UW-4 nanoparticles, to a glass vial placed on the stirring plate at 600 rpm, 1.0 mL of a solution of aqueous PVA (31 kDa) (1% *w*/*w*) was added, and the mixture was stirred (600 rpm). Then, five times (one portion per 1 min), 10.0 μL of RBOE solution in THF (1.0 mg/mL) was added and the vial was left open for the next 10 min [29].

Various WUT-type particles have been examined in our previous studies, in which their sensing properties and optical responses were evaluated. From the previously investigated particles, WUT-1 (anion-selective), WUT-2 (cation-selective), WUT-3 (amine-sensitive) and WUT-4 (containing the metalloporphyrin InOEP-Cl) were selected for the present work because they exhibited the most distinct and diverse responses to the dietary supplements analyzed in this study. In the case of UW particles, different systems were employed to broaden the sensitivity of the output signal.

All measurements were performed in eight replicates using freshly prepared, independent batches of WUT/UW particles, with each replicate performed in a separate, independent well. The eight replicates were obtained by distributing aliquots of the same supplement type and respective particle suspension among eight separate wells. WUT-1, WUT-2, WUT-3 and WUT-4 particle suspensions were pipetted in volumes of 50 μL, 100 μL, 100 μL and 50 μL, respectively. The batch-to-batch reproducibility and particle size of the WUT microspheres have been characterized in previous studies [10,25,26]. For the UW-type particles, similar volumes of suspensions were pipetted: 100 μL, 50 μL, 50 μL and 100 μL for UW-1, UW-2, UW-3 and UW-4, respectively. The wells were topped up to 100 μL with deionized water if necessary.

The next step was to add 100 μL of a previously prepared dietary supplement solution to individual wells. Information about the dietary supplements is provided in Table 2. Dietary supplements were prepared by dissolving a dose (1 tablet) in 250 mL of deionized water in a volumetric flask. The flask containing the suspension was then placed in an ultrasonic bath for 20 min to facilitate the release of the API and all excipients. To ensure clarity, the solution was filtered through a syringe filter (PTFE, 0.2 μm). After tenfold dilution, 100 μL aliquots of the dietary supplements were added to microwells on a microtiter plate. For each microtiter plate, a different tablet for the preparation of the supplement solution was applied. UV–Vis absorbance and fluorescence intensity spectra were recorded. These spectra were also obtained for the supplements themselves for further signal correction.

### 2.3. Apparatus

An ultrasonic bath (Sonic-0.5, 80 W, 40 kHz, POLSONIC Palczyński Sp. J., Warsaw, Poland) was used. All spectrophotometric and spectrofluorimetric measurements were performed with a Synergy MX multimode microplate reader (BioTek Instruments, Inc., Winooski, VT, USA) using Greiner CELLSTAR^®^ 96-well polystyrene microplates (Greiner Bio-One GmbH, Kremsmünster, Austria).

### 2.4. Data Analysis

All results were obtained as UV–Vis absorption spectra or fluorescence emission curves recorded at the appropriate excitation wavelengths (Table 1, Figure 1). To perform discriminant analysis and the classification of dietary supplements, the spectrum of the pure supplement sample was subtracted from each spectrum obtained for the mixture of the supplement sample and micro-/nanoparticles. The obtained data matrices containing the whole spectra for the respective supplements sets were then subjected to chemometric analyses to calculate the accuracy of the models: partial least squares analysis (PLS-DA), k-nearest neighbors (KNN), linear discriminant analysis (LDA), support vector machine analysis (SVM-DA) and Soft Independent Modeling of Class Analogy (SIMCA). To calculate the log F, point values were extracted from the spectral profiles, corresponding to the absorbance at the peak maximum and the fluorescence intensity at the emission maximum. Accuracy was calculated according to the formula presented in Table S1 in the Supplementary Information.

Prior to chemometric modeling, the data were mean-centered. For PLS-DA, the means of the individual variables were calculated from the training set and subsequently applied to both the training and test sets. The number of latent variables was selected based on the cross-validation results and RMSE minimization. For KNN, values of k ranging from 3 to 7 were evaluated. SVM-DA was performed using C-SVC models with a radial basis function (RBF) kernel. The cost and γ parameters were optimized using 5-fold cross-validation. For SIMCA, separate PCA models were constructed independently for each class, using one principal component per class. LDA was performed using the SVD algorithm. For all chemometric methods, model performance was evaluated using the training and test sets. All data analyses were performed using the Solo software 9.5(Eigenvector Research, Inc., Manson, WA, USA), and figures were generated in MS Excel 2020 (Microsoft, Redmond, WA, USA), JASP (https://jasp-stats.org/, accessed on 1 April 2026) and Origin 9.8.5.212 (OriginLab Corporation, Northampton, MA, USA).

## 3. Results and Discussion

A common issue in performing tests evaluating sensor usefulness for differential sensing—e.g., smart tongue/nose systems—is applying them for only a single, specific set of samples. This can lead to misleading conclusions regarding the sensor’s universality. To overcome this, this work focused on testing sensor performance across three sample sets, all composed of dietary supplements.

The first sample set (SET 1, Table 2) comprised five dietary supplements based on L-tyrosine-related biomolecules, targeting catecholamine metabolism, including N-acetyl-tyrosine, L-tyrosine and L-DOPA (neurotransmitter precursors). Two products contained free compounds—L-tyrosine and its acetylated form—facilitating better solubility and bioavailability (N-acetyl-L-tyrosine). The remaining three supplements were based on *Mucuna pruriens* seed extracts standardized to different L-DOPA content. The following sample set (SET 2, Table 2) comprised five neuromodulatory-action products based on complex mixtures of amino acids and derivatives (including L-glutamine, taurine and GABA), B vitamins and trace elements (B3, B5, B6, B9, chromium), diverse plant extracts and phytochemicals (such as ashwagandha, ephedra, nettle, guarana, white willow and yohimbe) and additional bioactive compounds including, e.g., inositol, choline bitartrate, naringin and phenylethylamine. Thus, these two independent sets presented different characteristics in terms of their discrimination. The first set comprised dietary supplements containing bioactive ingredients with very similar chemical structures, originating from both synthetic sources and biological materials, while, in contrast, the second set consisted mainly of supplements with complex compositions, in which the bioactive compounds were structurally diverse but unified by their analogous neuromodulatory effects. The third set was formed by the combination of SET 1 and SET 2 (SET 3, Table 2), i.e., the number of sample types was increased (10 instead of five), imposing higher demands on the discrimination process, because, with the expansion of the sample set, reliable discrimination becomes more challenging. To ensure the reliability of the obtained observations, we employed a triple random sampling method within each sample pool, as well as always dividing the data into train and test sets (five and three replicates, respectively). By calculating parameters such as the mean and standard deviation of each calculated parameter (log F, accuracy) for these three sample sets, the stability, repeatability and consistency of the results were rigorously assessed.

### 3.1. The Log F Parameter as a Measure of Pointwise Discriminatory Ability

The log F coefficient is a useful and versatile statistical measure that can be applied in smart sensing systems to quantitatively assess the discriminatory power of individual sensors. It originates from the Fisher–Snedecor F test, comparing variances in two or more groups, and is defined as the ratio of between-class variance to within-class variance; this enables the identification of sensors that provide unique and reproducible responses to different sample classes (Table S1) [30]. The log F has been applied for the identification of sensors with the highest discriminatory power in the classification of selected sample sets for sensor array reduction, allowing the elimination of redundant or highly correlated sensors without compromising analytical performance. It was shown that such a strategy improved the data quality, increased the variance explained by the principal components, enhanced sample separation in the PCA score plot and resulted in improved classification accuracy using artificial neural networks [30]. However, this measure prefers classes to be spread around the center/mean of the features and does not take into account their pairwise separability in multiclass problems. An analysis based on the log F coefficient also demonstrated that different sensors exhibit optimal performance depending on the analytical task and sample type, highlighting the context-dependent nature of sensor usefulness [31]. Thus, log F-based sensor selection leads to more efficient, robust and interpretable sensing systems; however, it should be treated as a complementary, rather than standalone, criterion for sensor selection.

**Figure 2:**
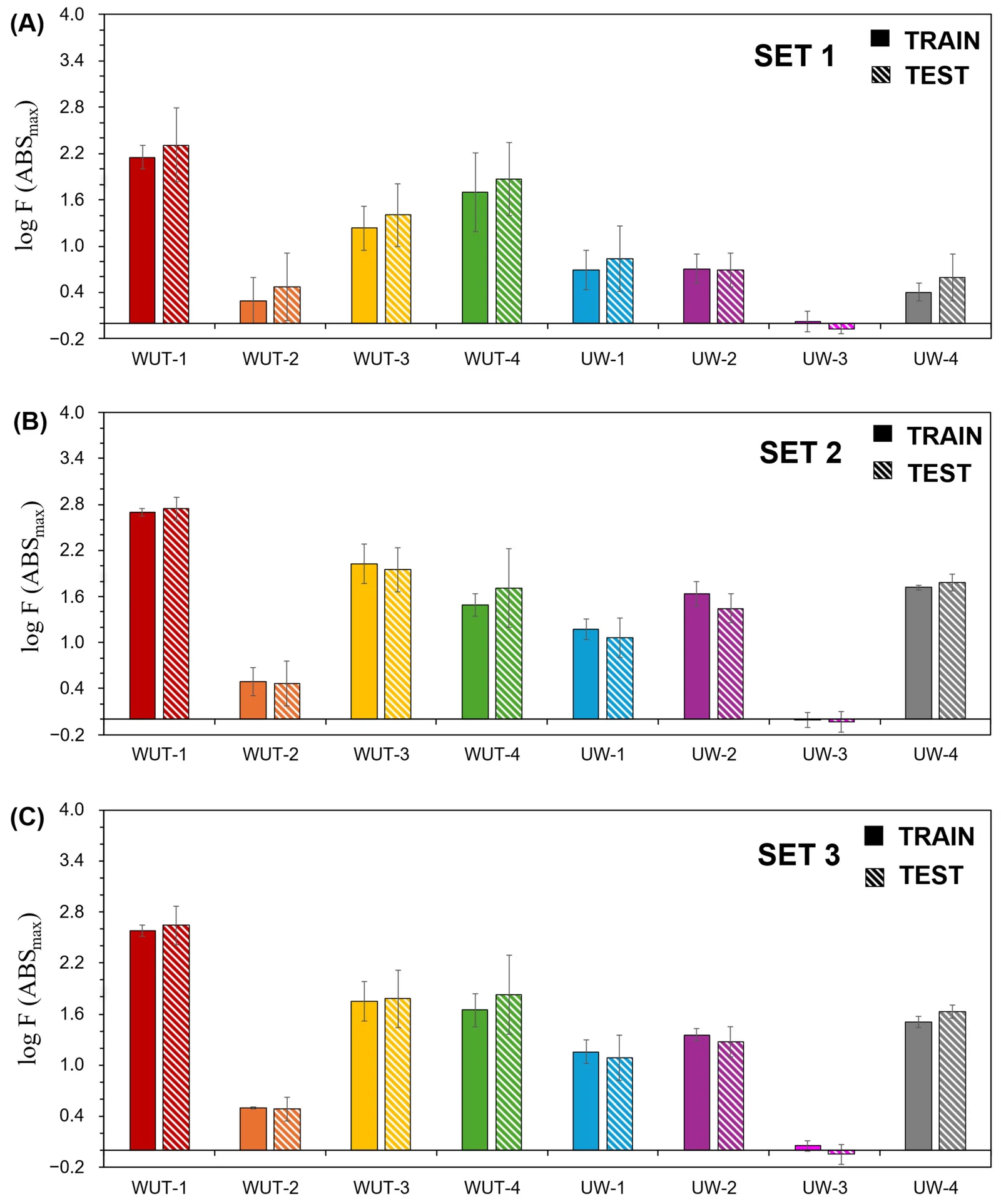

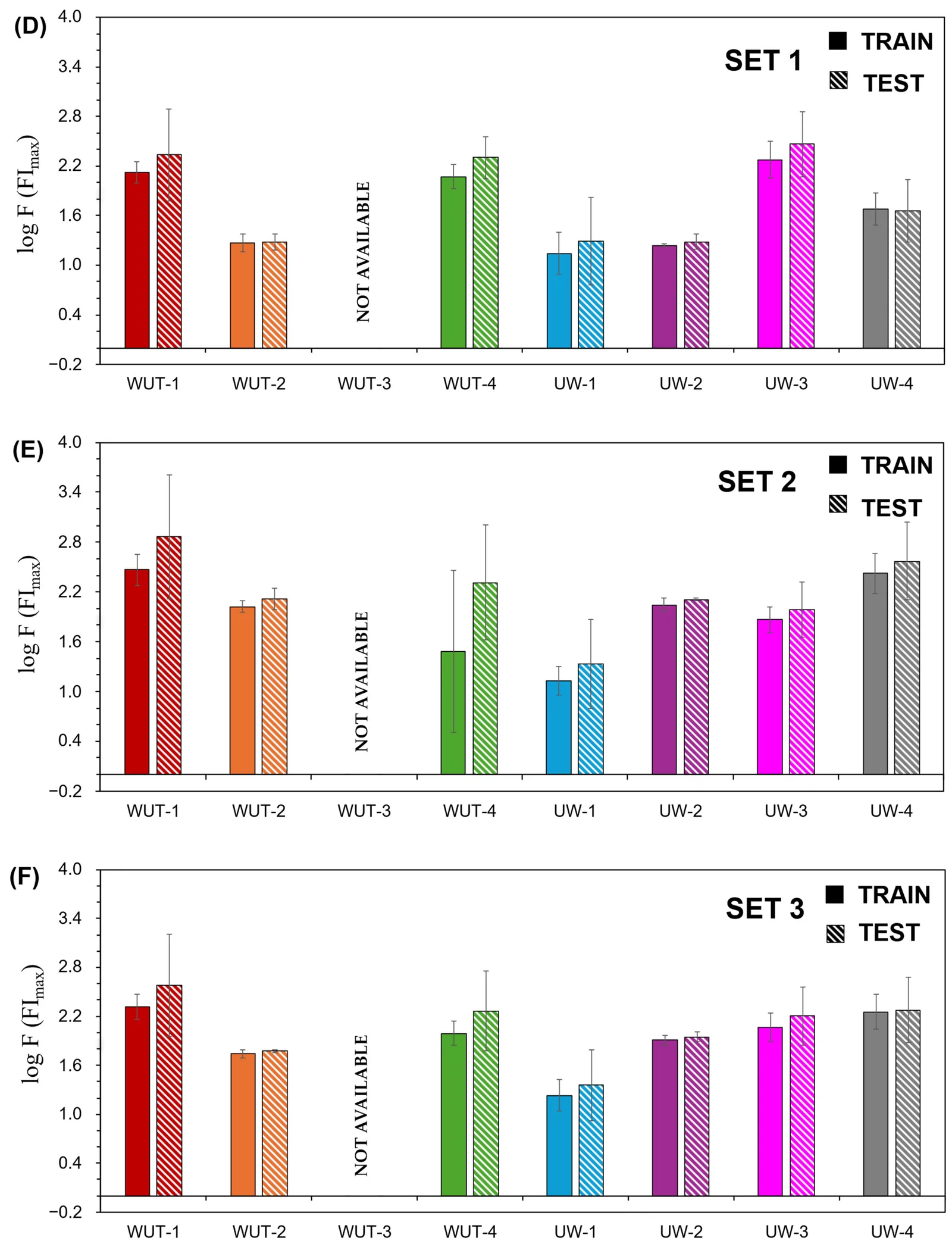
Log F values for three supplement sets (SET 1, SET 2 and combined SET 3, Table 2) based on pointwise signals: UV–Vis absorbance (**A**–**C**) and fluorescence intensity (**D**–**F**) data for train and test sets obtained using eight particle types (Table 1 and Figure S1, Supporting Information). Columns represent mean ± SD; *n* = 3 (numerical values of log F are provided in Table S2, Supporting Information).

The log F values were determined individually for the eight particle types based on the absorbance at the corresponding absorption maximum or the fluorescence intensity at the emission maximum. Figure 1 presents these values obtained for the three sets of supplements; the analyses were performed in parallel for the absorbance (Figure 2A–C) and fluorescence intensity signals (Figure 2D–F). It must be emphasized that an F value close to 1, i.e., log F close to 0, indicates poor discrimination between sample groups, whereas an F value exceeding the critical Fisher–Snedecor threshold confirms statistically significant differences between at least two groups. Thus, high log F values reflect the strong discriminative power of a sensor, i.e., they indicate the ability to distinguish multiple sample classes simultaneously, although they do not guarantee equally effective discrimination for every pair of sample types.

For UV–Vis measurements (Figure 2A–C), all particle types showed log F values above 0.2, indicating a certain degree of discriminative ability. A similar pattern of discriminative performance was consistently observed across all three sample sets. Only the UW-3 particles showed log F values close to zero, corresponding to a lack of discriminative capability in all studied datasets due to their fluorescence mode of response (Figure 2 [28]). In each sample set, the highest log F values were recorded for WUT-1 particles, with consistently high values also observed for WUT-3, WUT-4, UW-2 and UW-4. Lower but still satisfactory log F values were obtained for WUT-2 and UW-1, suggesting slightly limited but non-negligible discriminative performance.

In the case of fluorescence measurements (Figure 2D–F), the highest discriminative performance was observed again for WUT-1, along with the WUT-4, UW-3 and UW-4 particles—all of them reached values of log F > 2. The most pronounced change was noted for UW-3, which showed the strongest discrimination in fluorescence mode while being the weakest performer in the UV–Vis measurements. In contrast, WUT-1 particles no longer exhibited the highest log F values exclusively but reached discrimination levels comparable to those of the best-performing particles. Slightly lower log F values were observed for WUT-2, UW-1 and UW-2; however, it must be noted that WUT-2 and UW-2 displayed clearly improved discrimination when measured in fluorescence mode.

Importantly, as in the case of spectrophotometric measurements, the general patterns of discriminative performance for individual particle types were largely reproduced across all three datasets, despite the varying sizes of them. Even though similar response trends were observed in all datasets in both measurement modes (UV–Vis and fluorescence), these patterns were not identical. Consequently, particles identified as optimal within one dataset would not be the best performers in the case of another sample set. Furthermore, the discriminative abilities of individual particle types differed clearly between measurement modes, indicating that some particles are better suited for spectrophotometric detection, whereas others perform more effectively in fluorescence-based sensing, which supports the strategy of using multimode optical signals for enhanced performance in smart sensing systems.

Nevertheless, the F coefficient proved to be a robust and reliable tool for the quantitative assessment of discriminative capabilities. Notably, the analysis also demonstrated that most of the investigated particles could be used to construct a so-called virtual sensor array, exhibiting relatively high discrimination efficiency, enabling the differentiation of some sample types using only a single particle type. Finally, the comparison between the WUT 1–4 and UW 1–4 particles did not yield a straightforward conclusion regarding their overall superiority. Generally, particles designed for differential sensing, relevant to smart tongue applications, appeared more effective in spectrophotometric measurements. However, fluorescence measurements revealed comparable discrimination performance between particles engineered to facilitate cross-sensitive behavior (WUT) and those originally developed for standard, selective analyte detection (UW). This finding highlights the benefit of incorporating selectively responsive sensors, which are traditionally used for single-analyte determination, into sensor arrays, substantially expanding the pool of available sensing elements for smart tongue/nose sensing applications.

### 3.2. PLS-DA Classification Accuracy as a Measure of Full-Spectrum Discriminatory Ability

The accuracy parameter calculated using PLS-DA models provides significant information about the ability of particles to classify dietary supplements. Compared to log F analysis, which describes the differences in pointwise signals recorded by individual particles, the accuracy of chemometric models allows us to verify whether the obtained responses translate into a realistic ability to construct a model that can classify supplements statistically and predictively based on full-spectrum fingerprints (i.e., multiplexed signals). Therefore, the two approaches are complementary: the log F indicates potentially promising particles, while PLS-DA allows us to assess their utility in multivariate classification.

**Figure 3:**
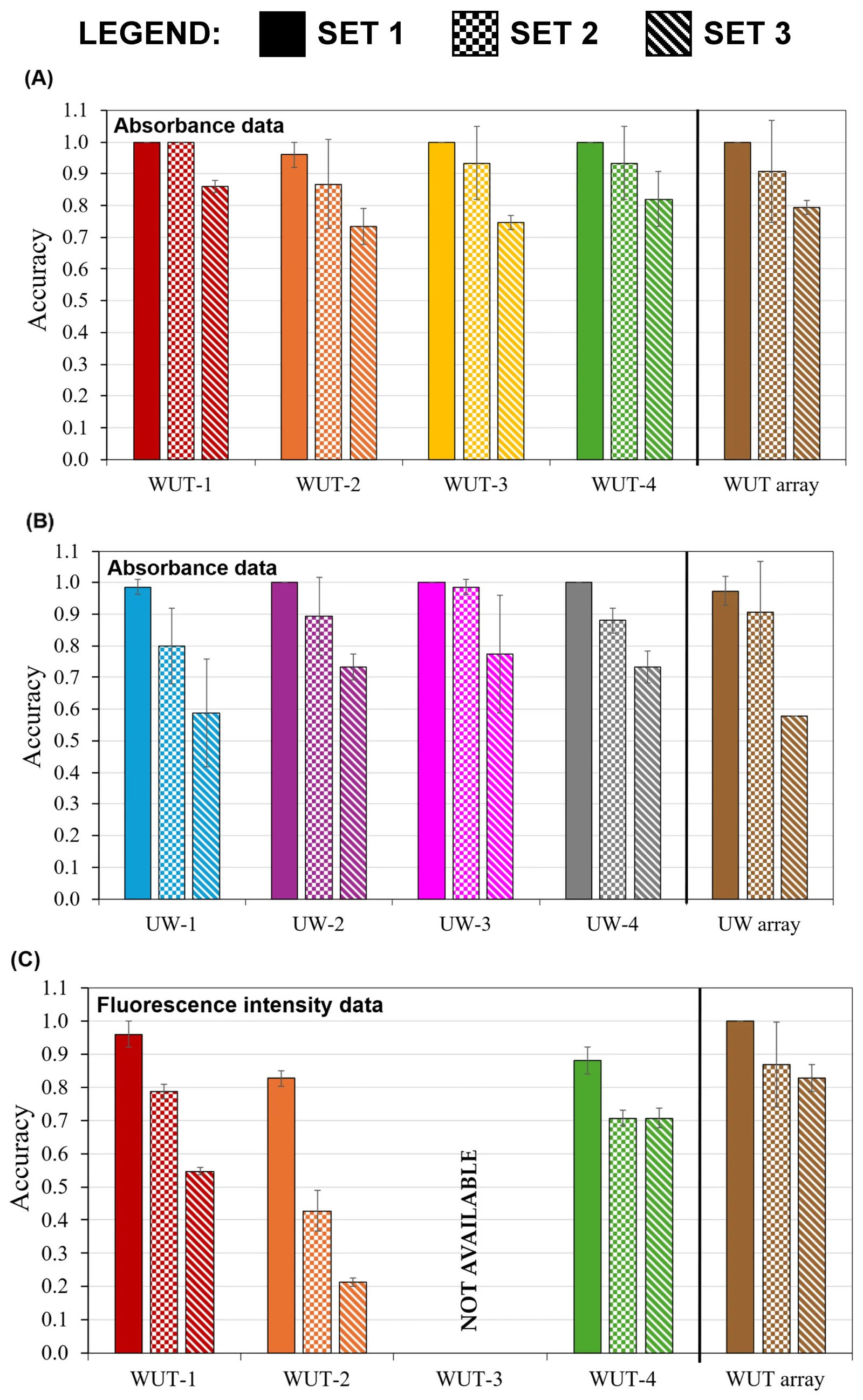

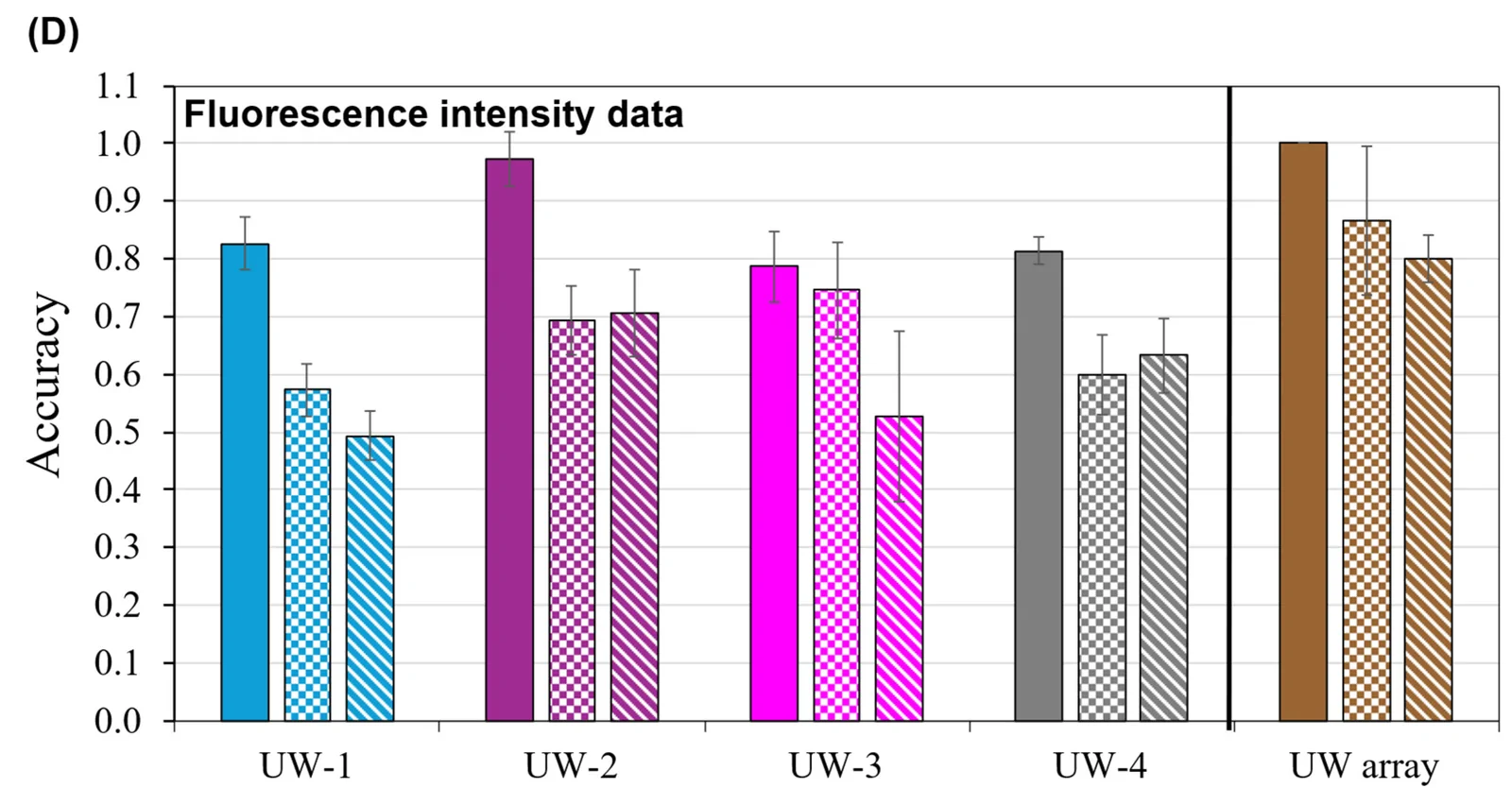
Classification accuracy of PLS-DA models calculated for three supplement sets (SET 1, SET 2 and combined SET 3, Table 2) based on full spectra: UV–Vis absorbance (**A**,**B**) and fluorescence intensity (**C**,**D**) data for the training sets, obtained using eight types of particles (Table 1). Columns represent mean ± SD; *n* = 3.

The data were divided into training and test sets. This division aimed to assess the reproducibility of the chemometric models, i.e., verifying whether the obtained discrimination between supplements was not solely due to fitting to the training set but remained consistent for the test data (i.e., the models were not overtrained). We used the full spectra recorded for all particles in both detection modes to evaluate the classification using PLS-DA. An analysis of the results shows that the classification accuracy varied depending on the particle type and the detection mode (Figure 3, Figure S2). Overall, the differences in classification accuracy between individual particles are less pronounced than those observed for the log F values (Figure 3). Importantly, the classification trends identified for the test datasets were consistently mirrored in the training sets, demonstrating consistent model performance between the training and test sets (Figure S2). For both the fluorescence and UV–Vis absorption measurements, and for all particles, a consistent trend toward the highest discrimination performance for sample SET 1 can be observed. In contrast, sample SET 2 exhibits reduced classification accuracy, while the most compositionally complex SET 3 consistently yields the lowest accuracy in both UV–Vis and fluorescence modes, which can be related to it having the highest number of classes.

In general, the classification accuracies obtained from the fluorescence measurements are lower than those derived from the UV–Vis data. For SET 1, very high accuracies were achieved, frequently reaching 100%. In UV–Vis mode, all particles provided perfect classification accuracy for this dataset. For WUT particles, similarly high accuracies were maintained for SET 2 (Figure 3A), whereas, for UW particles, the accuracy decreased to approximately 0.8 or below (Figure 3B). In the UV–Vis absorption measurements, no single best-performing particle can be clearly identified. All WUT particles exhibit comparable behavior, providing perfect classification performance for the training dataset, while UW particles also perform similarly, albeit slightly less effectively than the WUT type. The initially high—and, in some cases, perfect—classification abilities of individual particles result in similarly strong data fusion performance when particle arrays are constructed, regardless of whether WUT or UW particles are used (Figure 3A,B). Table S3A,B present the confusion matrices for the training and test sets, respectively, for the WUT array (SET 3), providing a detailed breakdown of the classification results.

In fluorescence mode, the classification performance becomes more differentiated among particles. Particularly for SET 2, lower accuracies were observed, dropping below 0.5 for WUT-2, and, for SET 3, the performance further decreased, reaching values as low as 0.2 for the same particle type. A recurring trend of the poorest classification performance for SET 3 is evident. Among all particles, WUT-1 consistently exhibits the highest classification ability, while the remaining WUT particles and all UW particles show similar, slightly lower performance. Importantly, a clear benefit of data fusion is observed, as the classification accuracies obtained for both the WUT and UW particle arrays exceed those achieved by the individual particles (Figure 3C,D).

To further evaluate the effects of data fusion, we combined the fluorescence data obtained from cross-sensitive and selective particles (WUT + UW array), and the results are presented in Table S4 (Supporting Information). For SET 1, the combined WUT + UW sensor array achieved classification accuracies of 0.99 for the training set and 0.98 for the test set, whereas the values for the individual WUT and UW arrays were 0.87 and 0.89, respectively. These results indicate that combining cross-sensitive and selective elements provided complementary information and improved the classification performance compared to the individual arrays.

**Figure 4:**
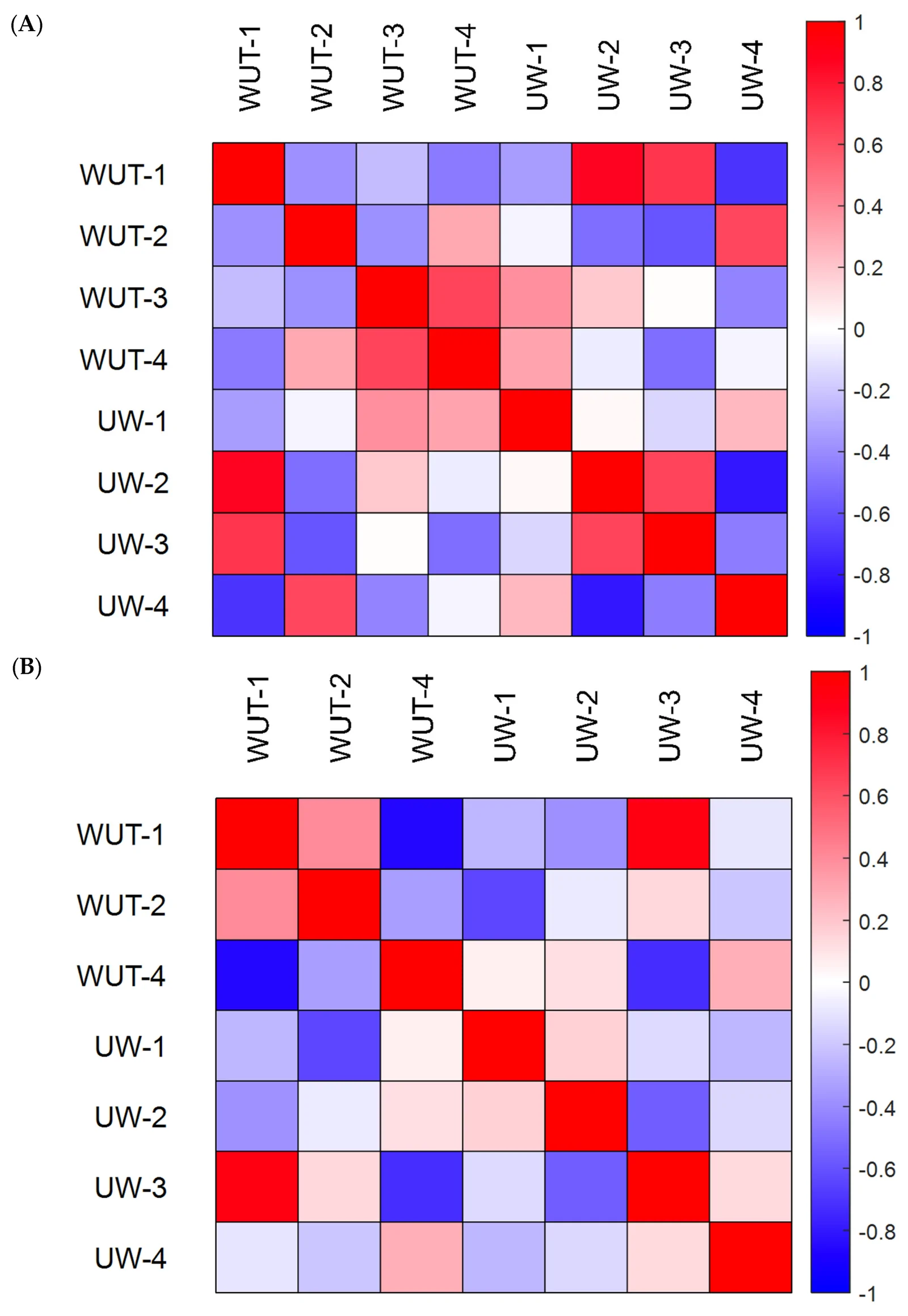
Correlation maps for pointwise signals of all particle types, illustrating the similarity of their responses across all studied samples: (**A**) absorbance data; (**B**) fluorescence intensity data.

The high classification accuracy achieved in the data fusion approach can be attributed to the complementary information provided by data sources exhibiting low inter-particle correlations (reduction of redundancy). To demonstrate this hypothesis, correlation maps for all particle types, illustrating the similarity of their responses across all studied samples, are presented in Figure 4. For the absorbance data, the lowest correlations (i.e., Pearson correlation coefficients close to zero) were observed for the following particle pairs: UW-3 and WUT-3; UW-4 and WUT-4; UW-1 and UW-2; and UW-1 and WUT-2 (Figure 4A). In the case of the fluorescence data, the lowest correlation was observed for the UW-1–WUT-4 pair (Figure 4B). These findings support the advantage of the “smart tongue” approach, which integrates diverse particle types, both selective and cross-sensitive, to enhance the complementarity of the acquired responses.

Table S3C,D present the confusion matrices for the training and test sets, respectively, for the UW system (SET 3), providing a detailed breakdown of the classification results. To further evaluate the effects of the multimode strategy, the absorbance and fluorescence data were combined into a single ABS + FI model. For SET 1, using WUT particles, the classification accuracy for the training set was 0.91, 0.87 and 0.93 for the absorbance, fluorescence intensity and combined ABS + FI data, respectively. For the test set, the corresponding accuracies were 0.89, 0.89 and 0.93, respectively. The corresponding results are summarized in Supplementary Table S5. These results indicate that the integration of absorbance and fluorescence data (i.e., a multimode strategy) provided complementary information and improved the classification performance compared with the individual detection modes.

A comparison of the log F-based evaluation (Table S2, Figure 2) and full-spectrum classification performance (Figure 3) reveals both consistent trends and notable differences. In both approaches, SET 1 is the easiest to discriminate, consistently yielding the highest performance, whereas the most compositionally complex SET 3 exhibits the poorest discrimination or classification results. This agreement confirms that increasing sample complexity (number of classes) negatively affects system performance regardless of whether pointwise (log F) or full-spectrum analysis is applied. However, important discrepancies are observed at the level of individual particles. While the log F analysis highlights pronounced differences in discriminative power among particles and clearly differentiates between strong and weak performers depending on the measurement mode, these differences are substantially attenuated in the full-spectrum approach. In the UV–Vis measurements, particles exhibiting markedly different log F values achieve similarly high, often perfect classification accuracies when the entire spectral fingerprints are used, indicating that information distributed across the spectrum complements the limited pointwise discrimination, which supports the strategy of using multiplexed optical signals for enhanced performance in smart sensing systems.

In the fluorescence mode, partial agreement between the two approaches is retained: particles identified by log F as weak discriminators, such as WUT-2, also show reduced full-spectrum classification accuracy, particularly for SET 2 and SET 3. Nevertheless, the magnitude of the performance differences between particles remains smaller than suggested by the log F values alone. Overall, while the log F effectively captures the intrinsic discriminative sensitivity of individual particles, full-spectrum analysis demonstrates greater robustness by exploiting multidimensional spectral information, thereby reducing the performance disparities observed in the pointwise evaluation.

### 3.3. Comparison of Classification Accuracy of Various Chemometric Methods

In the previous section, we highlight PLS-DA as the gold standard for high-dimensional, highly collinear, full-spectrum data, especially suitable for smart sensing. It serves as a baseline supervised classification method against which other approaches can be compared. In order to determine whether the previous findings were reproducible across various data analysis methods or were artifacts of the chosen benchmark, we employed KNN, LDA, SVM-DA and SIMCA for the investigation of the optical signals of “smart tongues” formed by the cross-sensitive (WUT array) and selective (UW array) sensors used for the recognition of samples included in SET 3. These five classification methods were selected as they represent complementary modeling strategies; differ in their assumptions, interpretability and robustness to data correlation; and are widely used in the chemometric analysis of complex optical sensor responses. PLS-DA and LDA are linear projection-based methods that offer high interpretability but may be limited when class boundaries are non-linear, whereas KNN and SVM can capture more complex data structures at the expense of reduced model transparency. In contrast, SIMCA follows a class-modeling strategy, enabling the assessment of class membership and outlier detection, rather than direct multiclass recognition [32,33,34].

PLS-DA is a supervised method used for dimensionality reduction. It takes into account class labels to maximize the separation between groups. This means that the data transformation in PLS-DA is guided by the variability between groups, allowing for the better visualization of differences between sample classes in the graph. This makes PLS-DA particularly useful for spectral fingerprinting analysis, where the goal is to distinguish between previously defined experimental groups [35,36]. The group of classical linear methods also includes LDA. Similarly to PLS-DA, it transforms the data into a new space and maximizes the ratio of interclass to intraclass variance. As a result, LDA enables the more efficient classification and visualization of differences between samples than unsupervised methods such as PCA [37]. An alternative to linear methods is the KNN algorithm, which classifies samples based on their similarity to the objects closest in the feature space. The most commonly used metric is the Euclidean distance, and the optimal number of neighbors is selected using leave-one-out cross-validation. As a result, KNN does not require a global model or assumptions about the shape of class boundaries, making it particularly effective for data with partial class overlap [38,39]. Another method offering greater classification capabilities is SIMCA. Unlike KNN, which relies on the proximity of samples, SIMCA builds a separate model for each class based on PCA, which describes the shape and position of samples in a multidimensional space. New samples are assigned to a class if they fall within its designated area, which additionally allows for the identification of outliers. This method works well when classes have a complex structure and are not easily separable linearly [39,40]. The most advanced approach among the methods discussed is SVM, which can be implemented either as a linear model or with kernel functions to handle non-linear class boundaries. Non-linear SVM handles non-linear dependencies and ensures high classification stability. Furthermore, owing to the principle of structural risk minimization, this method reduces the risk of overfitting and often outperforms classical approaches in chemometrics, especially when the differences between classes are minor [32,33,41,42].

**Figure 5:**
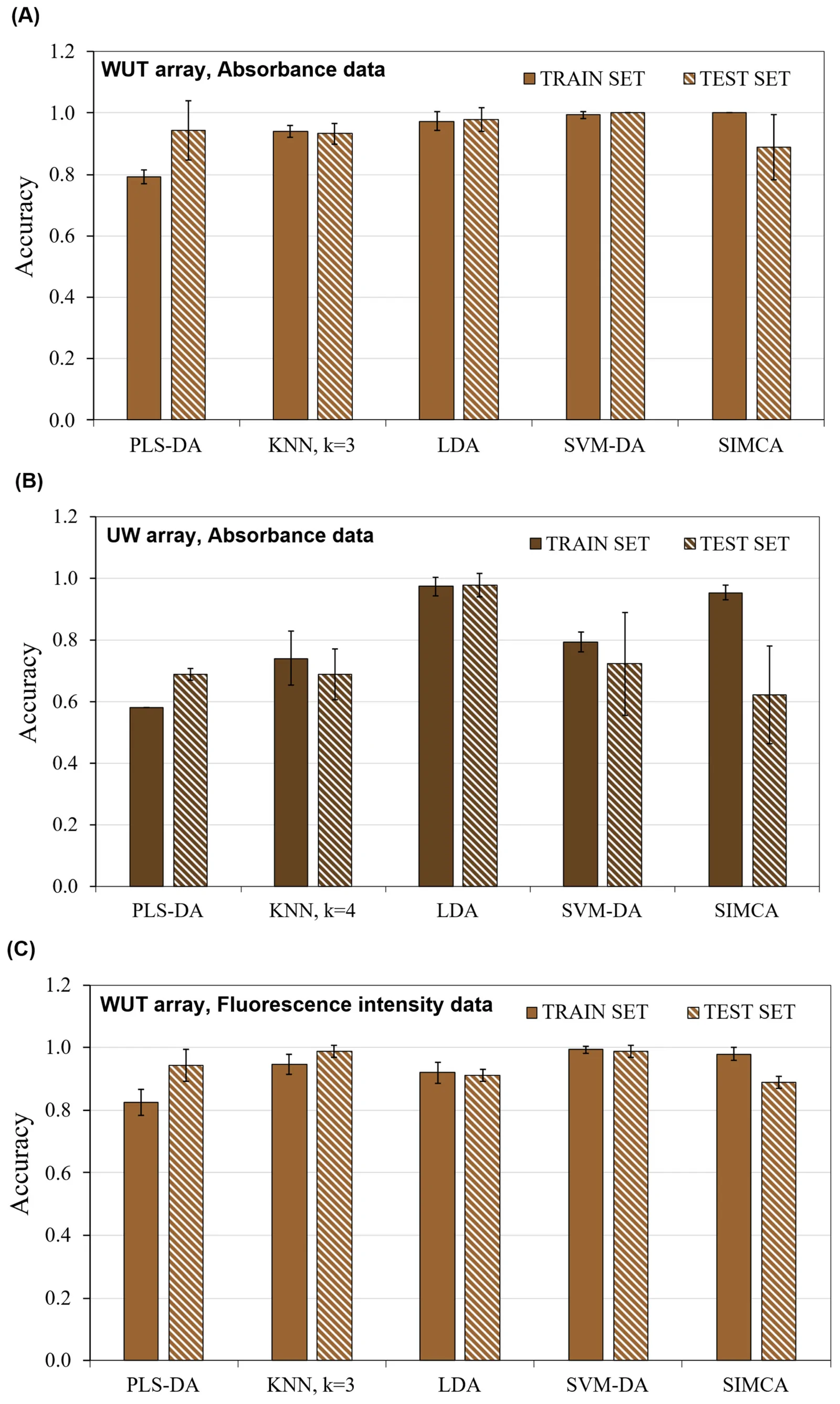

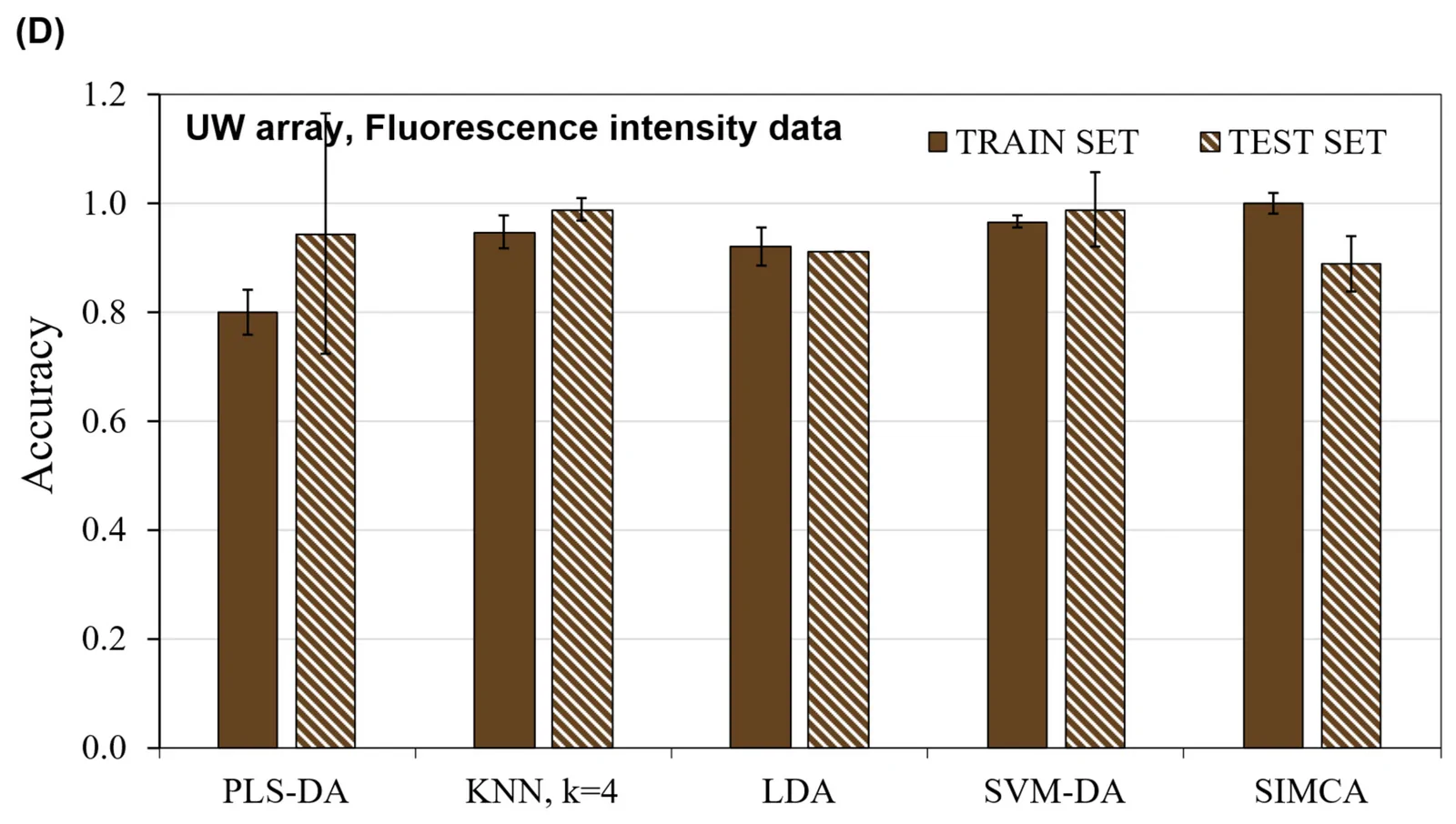
Comparison of the accuracy of 5 classification models, PLS-DA, KNN, LDA, SVM-DA and SIMCA, in the analysis of SET 3 dietary supplements based on UV–Vis absorbance (**A**,**B**) and fluorescence intensity (**C**,**D**) data. Columns represent mean ± SD; *n* = 3. The corresponding numerical data are provided in the Supporting Information, Table S6.

The comparative analysis of the classification models (PLS-DA, KNN, LDA, SVM-DA, SIMCA) applied to the absorbance and fluorescence data revealed significant differences in their ability to distinguish the dietary supplements included in SET 3. For WUT particles (Figure 5A,C), the classic PLS-DA model achieved moderate accuracy (approximately 0.79–0.94). At the same time, non-linear SVM-DA provided significantly higher stability and better results in the training and test sets (>0.94). These methods proved particularly useful for the fluorescence data, where PLS-DA previously showed limitations (e.g., WUT-2). It is worth noting that both KNN (k = 3) and LDA provided robust results, although their performance was more dependent on the input data type: LDA performed well with UV–Vis absorption data; in contrast, KNN performed well with fluorescence intensity data. Additional analysis (Figure S3A,C) indicates that the choice of parameter k in KNN was significant for WUT particles. With k = 4, the accuracy increased in some cases. Still, a comprehensive analysis, including both detection modes and training and test sets, shows that more stable and generally better results were obtained with k = 3. This justifies the choice of this parameter value for the overall comparison with other methods.

For UW particles (Figure 5B,D), greater variability was observed between the measurement modes, especially for the absorbance data. PLS-DA underperformed compared to the other techniques (accuracy ~0.6–0.7), confirming its limited ability to capture more complex patterns. LDA provided the highest accuracy (>0.9) and was also characterized by good generalization between the training and test sets. In the case of fluorescence measurements, the UW array yielded more consistent results, and the best results were again obtained for non-linear SVM-DA and KNN, while PLS-DA remained at a lower level. In this case, KNN (k = 4) performed better with spectrofluorimetric responses than spectrophotometric ones, and changing the k value from 3 to 7 (Figure S3B,D) did not lead to significant differences in accuracy. The results were relatively stable, albeit lower than those for the best chemometric models.

The comparison clearly indicates that, while PLS-DA is a versatile method and allows for easy interpretation through dimensionality reduction, its performance can be improved, especially for fluorescence data. The use of other techniques, such as SVM-DA, allowed for achieving higher accuracy and stability; at the same time, it is better able to handle multiclass problems (SET 3).

## 4. Conclusions

A significant challenge in smart sensing is the rational selection of sensing elements to ensure universality and robustness. Selective sensors perform well under controlled conditions but may be limited in more complex, real samples, while cross-sensitive sensors enable the simultaneous detection of multiple analytes at the cost of more complex calibration and signal interpretation. This work investigated three complementary strategies: reducing redundancy through lower response correlation, increasing information content using full spectral fingerprints and exploiting multimode optical readouts.

Three dietary supplement datasets were analyzed to evaluate sensor universality. Pointwise discrimination was assessed using the log F coefficient, while full-spectrum fingerprint performance was evaluated through supervised chemometric classification. The results showed that sensor effectiveness depends on the measurement mode and sample set, enabling the identification of task-specific leading sensors (e.g., WUT-1 and WUT-4 for SET 1 spectrophotometric measurements; WUT-1 and UW-3 for SET 1 fluorescence intensity measurements). Consistent patterns across datasets confirmed the robustness of the log F metric.

Full-spectrum analysis using PLS-DA led to satisfactory classification outputs, while similar training and test results indicated the absence of overfitting. Data fusion further improved the classification accuracy. The comparison of PLS-DA, LDA, KNN, SIMCA and SVM-DA demonstrated that, while linear methods offered interpretability, non-linear approaches, particularly SVM-DA, provided superior accuracy and stability by effectively capturing subtle, non-linear class differences.

The above results demonstrate that combining selective and cross-sensitive sensors, multimode optical readout and appropriate multivariate analysis enables the construction of flexible, information-rich and robust smart sensing platforms suitable for complex analytical problems. Still, this work explored a limited number of samples and sample sets, as well as specific types of sensors (optical chemosensitive micro-/nanoparticles); therefore, future analogues studies considering other experimental set-ups should be undertaken to validate the generalizability of these findings.

## Figures and Tables

**Table 1:** The composition of components used for the fabrication of the developed micro- and nanoparticles (WUT and UW, respectively) together with their basic spectrophotometric (λ_max_) and spectrofluorimetric (λ_ex_/λ_em_) properties.

Particle ID	Surfactant	Plasticizer	Ion Exchanger	Chromoionophore	Ionophore	Optical Properties
**WUT-1**	F127, 12.5 mg	DOS, 4 mg	TDMAC, 0.59 mg	ETH 7061, 0.6 mg	-	λ_max_ = 454 nm λ_ex_/λ_em_ = 463 nm/555 nm
**WUT-2**		DOS, 4 mg	KTFPB, 1.55 mg	ETH 5294, 0.5 mg	-	λ_max_ = 610 nm λ_ex_/λ_em_ = 614 nm/684 nm
**WUT-3**		o-NPOE, 4 mg	NaTFPB, 1.2 mg	ETH 2439, 0.47 mg	Lasalocid A sodium salt, 2.18 mg	λ_max_ = 666 nm
**WUT-4**	Triton X-100, 12.5 mg	o-NPOE, 4 mg	KTFPB, 0.30 mg	-	InOEPCl, 1.9 mg	λ_max_ = 406 nm λ_ex_/λ_em_ = 406 nm/576 nm
**UW-1**	PVA, 50 mg	-	NaTFPB, 2.40 mg	PHT-COOH, 1 mg	ETH1001, 6.93 mg	λ_max_ = 516 nm λ_ex_/λ_em_ = 540 nm/636 nm
**UW-2**	PVA, 50 mg	-	-	PHT-COOH, 1 mg	-	λ_max_ = 522 nm λ_ex_/λ_em_ = 487 nm/623 nm
**UW-3**	PVA, 10 mg	DOS, 15 mg	NaTFPB, 1 mg	POT, 1 mg	-	λ_max_ = 564 nm λ_ex_/λ_em_ = 550 nm/736 nm
**UW-4**	PVA, 10 mg	-	-	RBOE, 0.01 mg	-	λ_max_ = 570 nm λ_ex_/λ_em_ = 550 nm/610 nm

**Table 2:** Specifications of the tested supplement sets: SET 1, composed of 5 supplements with L-tyrosine-related neuroactive precursors, and SET 2, composed of 5 neuromodulatory supplements. SET 3 is the combined set of all 10 supplements.

	No.	Type of Supplement	Specification
**SET 1**	1	N-acetyl-tyrosine Allines Health ‘n Beauty	N-acetyl-tyrosine (500 mg)
	2	FORTE L-TYROSINE Progress Labs	L-tyrosine (500 mg)
	3	L-DOPA 7NUTRITION	Mucuna pruriens extract 25% from seeds (580 mg), including L-DOPA (145 mg)
	4	L-DOPA Wish Pharmaceutical	Mucuna pruriens extract standardized to 98% L-DOPA (300 mg), Orafti^®^GR inulin (70 mg)
	5	L-DOPA Progress Labs	Mucuna pruriens extract standardized to 98% L-DOPA (300 mg), Orafti^®^GR inulin (70 mg)
**SET 2**	A	G BOMB	Micronized L-glutamine (1 g), inositol (500 mg), choline bitartrate (82.5 mg), ashwagandha extract preparation (130 mg), naringin (55 mg)
	B	Yellow Bullet	Ephedra extract, glucuronolactone, phenylethylamine HCL, synephrine HCL, nettle root extract, yohimbine HCL, xanthinol niacinate, picamilon, ephedra leaf extract
	C	GABA + B6	Vitamin B6—pyridoxine HCL (2 mg), GABA (500 mg)
	D	ECA ELITE	Vitamins B3, B5, B6, B9, chromium, caffeine anhydrous, glucuronolactone, Ephedra viridis, guarana seed, synephrine HCL, nettle root extract, white willow bark, yohimbe bark extract, xanthinol nicotinate
	E	Taurine	Taurine (500 mg)
**SET 3**	Supplements 1–5 and A–E		

## Data Availability

Data is contained within the article or Supplementary Material.

## Supplementary Materials

The following supporting information can be downloaded at: https://www.mdpi.com/article/10.3390/s26196312/s1. Table S1. Definitions of parameters applied to characterize discriminatory properties of the studied micro/nanoparticles (in the article log F was presented).; Table S2. Log F values for three supplement sets based on point-wise signals based on UV-Vis absorbance and fluorescence intensity measurements.; Table S3. Confusion matrices for PLS-DA models applied to the identification of dietary supplements from SET 3 using micro/nanoparticles: (A) results for the training set based on UV-Vis data for the WUT array; (B) results for the test set based on UV-Vis data for the WUT array; (C) results for the training set based on fluorescence data for the UW array; (D) results for the test set based on fluorescence data for the UW array.; Table S4. Classification accuracy of PLS-DA models for fluorescence intensity data for SET 1 obtained using the WUT array, UW array, and combined WUT+UW array (data fusion) for the training and test sets.; Table S5. Classification accuracy of PLS-DA models for SET 1 and WUT particles, based on absorbance, fluorescence intensity, and combined absorbance and fluorescence measurements (data fusion, ABS+FI).; Table S6. Comparison of the accuracy (mean value, *n* = 3) of 5 classification models: PLS-DA, KNN, LDA, SVM-DA, SIMCA, in the analysis of SET 3 dietary supplements based on UV-Vis absorbance and fluorescence intensity data.; Figure S1. Comparison of raw spectral readings for all particle types obtained for all dietary supplements, used in point-wise analysis for log F calculation. Values of absorbance at the corresponding absorption maximum (A) or the fluorescence intensity at the emission maximum (B) were presented in the form of a box-plot, where edges of the box represent Q1 and Q3; line represents median, and whiskers show 1.5IQR.; Figure S2. Classification accuracy of PLS-DA models calculated for three supplement sets (SET 1, SET 2, and combined SET 3, Table S2); based on full spectra: UV-Vis absorbance (A, B) and fluorescence intensity (C, D) data for the test sets, obtained using eight types of particles (Table S1). Columns represent mean ± SD; *n* = 3.; Figure S3. Comparison of the accuracy of KNN classification models in the analysis of SET 3 dietary supplements based on UV-Vis absorbance (A, B) and fluorescence intensity (C, D) data. Columns represent mean ± SD; *n* = 3.
