# Supplementary material for: Balancing Selectivity and Cross-Sensitivity in Smart Tongue Sensing—Multimode, Multiplexed Optical Sensor Arrays for Dietary Supplement Classification
Source: Sensors (Basel). 2026 Oct 6;26(19):6312. doi: 10.3390/s26196312 (PMC13667469; doi:10.3390/s26196312)
Supplement: Supplementary file 1 [file sensors-26-06312-s001.zip › sensors-4519371-supplementary.pdf]

Supporting Information

Table S1. Definitions of parameters applied to characterize discriminatory properties of the studied micro-/nanoparticles (in the main text, the log F is presented) [30,31].

$F=\dfrac{\dfrac{\sum_{i=1}^{k} n_i\left(\bar{z}_i-\bar{\bar{z}}\right)^2}{k-1}}{\dfrac{\sum_{i=1}^{k}\sum_{j=1}^{n_i}\left(z_{ji}-\bar{z}_i\right)^2}{\sum_{i=1}^{k} n_i-k}}$	Mean value of sensor response for all samples: $\bar{\bar{z}}=\dfrac{\sum_{i=1}^{k}\sum_{j=1}^{n_i} z_{ji}}{\sum_{i=1}^{k} n_i}$ Mean value of sensor response in a particular class of samples: $\bar{z}_i=\dfrac{\sum_{j=1}^{n_i} z_{ji}}{n_i}$ k—number of classes i—number of following class j—following number of samples in i-th class z_{ji}—sensor response for j-th sample in i-th class n_i—number of samples in i-th class
$accuracy=\dfrac{TP+TN}{TP+TN+FP+FN}$	TN—true negatives FN—false negatives FP—false positives TP—true positives

Table S2. Log F values for three supplement sets based on pointwise signals derived from UV–Vis absorbance and fluorescence intensity measurements.

		log F							
		ABSORBANCE							
		WUT particles				UW particles			
		WUT-1	WUT-2	WUT-3	WUT-4	UW-1	UW-2	UW-3	UW-4
SET 1	Train	2.15	0.30	1.23	1.70	0.69	0.71	0.02	0.40
	Test	2.31	0.47	1.40	1.87	0.84	0.70	-0.07	0.59
SET 2	Train	2.70	0.49	2.03	1.49	1.17	1.64	-0.01	1.72
	Test	2.74	0.47	1.95	1.71	1.07	1.45	-0.04	1.78
SET 3	Train	2.58	0.50	1.75	1.64	1.16	1.36	0.05	1.51
	Test	2.64	0.48	1.78	1.82	1.09	1.27	-0.05	1.62
		log F							
		FLUORESCENCE INTENSITY							
		WUT particles				UW particles			
		WUT-1	WUT-2	WUT-3	WUT-4	UW-1	UW-2	UW-3	UW-4
SET 1	Train	2.12	1.27	NA	2.07	1.14	1.24	2.28	1.68
	Test	2.34	1.28	NA	2.30	1.29	1.29	2.46	1.66
SET 2	Train	2.47	2.02	NA	1.48	1.13	2.04	1.87	2.42
	Test	2.86	2.11	NA	2.31	1.33	2.11	1.99	2.57
SET 3	Train	2.31	1.74	NA	1.99	1.23	1.90	2.06	2.25
	Test	2.58	1.77	NA	2.27	1.36	1.95	2.20	2.28

Table S3. Confusion matrices for PLS-DA models applied to the identification of dietary supplements from SET 3 using micro-/nanoparticles: (A) results for the training set based on UV–Vis data for the WUT array; (B) results for the test set based on UV–Vis data for the WUT array; (C) results for the training set based on fluorescence data for the UW array; (D) results for the test set based on fluorescence data for the UW array.

(A)

PREDICTED CLASS	REAL CLASS									
	**I**	**II**	**III**	**IV**	**V**	**VI**	**VII**	**VIII**	**IX**	**X**
**I**	5	0	0	0	0	0	0	0	0	0
**II**	0	5	0	0	0	0	0	0	0	0
**III**	0	0	5	0	0	0	0	0	0	0
**IV**	0	0	0	5	0	0	0	0	0	0
**V**	0	0	0	0	5	0	0	0	0	0
**VI**	0	0	0	0	0	5	0	0	0	0
**VII**	0	0	0	0	0	0	5	0	0	0
**VIII**	0	0	0	0	0	0	0	5	0	0
**IX**	0	0	0	0	0	0	0	0	5	2
**X**	0	0	0	0	0	0	0	0	0	3

(B)

PREDICTED CLASS	REAL CLASS									
	**I**	**II**	**III**	**IV**	**V**	**VI**	**VII**	**VIII**	**IX**	**X**
**I**	3	0	0	0	0	0	0	0	0	0
**II**	0	3	0	0	0	0	0	0	0	0
**III**	0	0	3	0	2	0	0	0	0	0
**IV**	0	0	0	3	0	0	0	0	0	0
**V**	0	0	0	0	1	0	0	0	0	0
**VI**	0	0	0	0	0	3	0	0	0	0
**VII**	0	0	0	0	0	0	3	0	0	0
**VIII**	0	0	0	0	0	0	0	3	0	0
**IX**	0	0	0	0	0	0	0	0	2	2
**X**	0	0	0	0	0	0	0	0	1	1

(C)

PREDICTED CLASS	REAL CLASS									
	**I**	**II**	**III**	**IV**	**V**	**VI**	**VII**	**VIII**	**IX**	**X**
**I**	5	0	0	0	0	0	0	0	0	0
**II**	0	5	0	0	0	0	0	0	0	0
**III**	0	0	2	0	0	0	0	0	0	0
**IV**	0	0	0	5	0	0	0	0	0	0
**V**	0	0	0	0	4	0	0	0	0	0
**VI**	0	0	0	0	0	5	0	0	0	0
**VII**	0	0	1	0	0	0	5	0	0	0
**VIII**	0	0	0	0	0	0	0	5	0	0
**IX**	0	0	0	0	0	0	0	0	4	1

**X**	0	0	2	0	1	0	0	0	1	4

(D)

REAL CLASS

PREDICTED CLASS	I	II	III	IV	V	VI	VII	VIII	IX	X
**I**	3	0	0	0	0	0	0	0	0	0
**II**	0	3	0	0	0	0	0	0	0	0
**III**	0	0	0	0	0	0	0	0	0	1
**IV**	0	0	0	3	0	0	0	0	0	0
**V**	0	0	0	0	2	0	0	0	1	0
**VI**	0	0	0	0	0	3	0	0	0	0
**VII**	0	0	2	0	0	0	3	0	0	0
**VIII**	0	0	0	0	0	0	0	3	0	0
**IX**	0	0	0	0	0	0	0	0	0	1
**X**	0	0	1	0	1	0	0	0	2	1

Table S4. Classification accuracy of PLS-DA models for fluorescence intensity data for SET 1 obtained using the WUT array, UW array and combined WUT+UW array (data fusion) for the training and test sets.

Fluorescence Intensity Data		ACCURACY		
		Particles		
		WUT array	UW array	WUT+UW array
SET 1	Train	0.87	0.87	0.99
	Test	0.89	0.89	0.98

Table S5. Classification accuracy of PLS-DA models for SET 1 and WUT particles, based on absorbance, fluorescence intensity and combined absorbance and fluorescence measurements (data fusion, ABS+FI).

		ACCURACY		
		WUT particles		
		Absorbance data	Fluorescence intensity data	ABS + FI
SET 1	Train	0.91	0.87	0.93
	Test	0.89	0.89	0.93

Table S6. Comparison of the accuracy (mean value, n=3) of 5 classification models, PLS-DA, KNN, LDA, SVM-DA and SIMCA, in the analysis of SET 3 dietary supplements based on UV–Vis absorbance and fluorescence intensity data.

		ACCURACY									
		ABSORBANCE DATA									
		WUT array					UW array				
		PLS-DA	KNN, k=3	LDA	SVM-DA	SIMCA	PLS-DA	KNN, k=4	LDA	SVM-DA	SIMCA
SET 3	Train	0.79	0.94	0.97	0.99	1.00	0.58	0.74	0.97	0.79	0.95
	Test	0.94	0.93	0.98	1.00	0.89	0.69	0.69	0.98	0.72	0.62
		ACCURACY									
		FLUORESCENCE INTENSITY DATA									
		WUT array					UW array				
		PLS-DA	KNN, k=3	LDA	SVM-DA	SIMCA	PLS-DA	KNN, k=4	LDA	SVM-DA	SIMCA
SET 3	Train	0.83	0.95	0.92	0.99	0.98	0.80	0.95	0.92	0.97	0.89
	Test	0.94	0.99	0.91	0.99	1.00	0.86	0.94	0.90	0.91	0.88

Figure S1. Comparison of raw spectral readings for all particle types obtained for all dietary supplements, used in pointwise analysis for log F calculation. Values of absorbance at the corresponding absorption maximum (A) or the fluorescence intensity at the emission maximum (B) are presented in the form of a box plot, where the edges of the box represent Q1 and Q3; lines represent the median; and whiskers show the 1.5 IQR.

A)

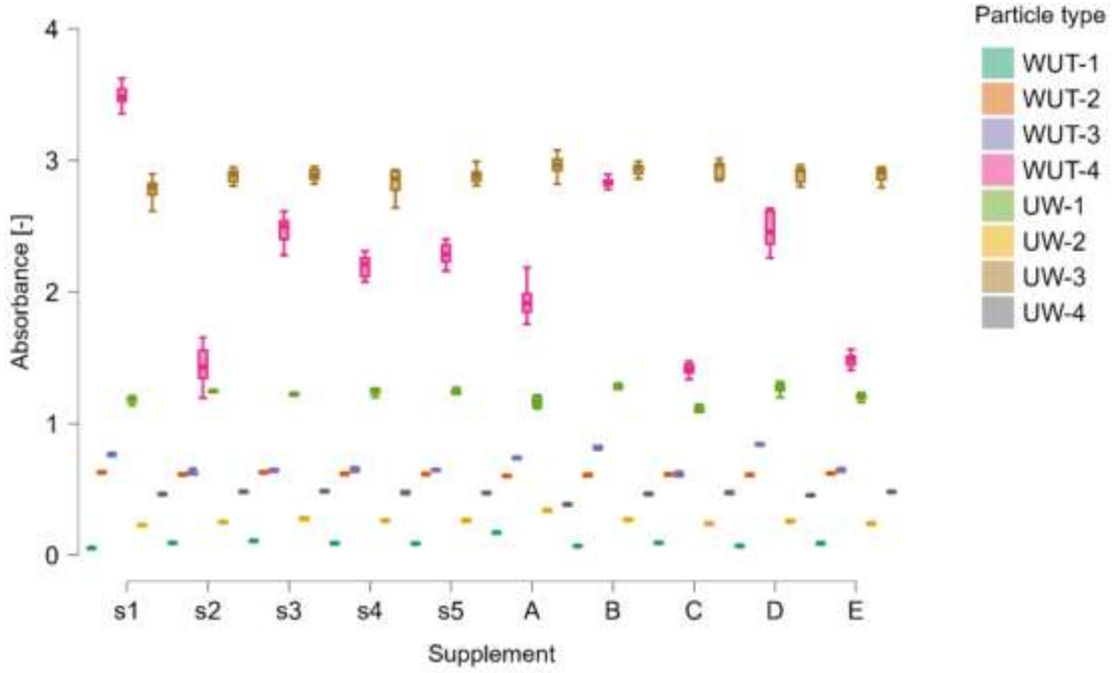

B)

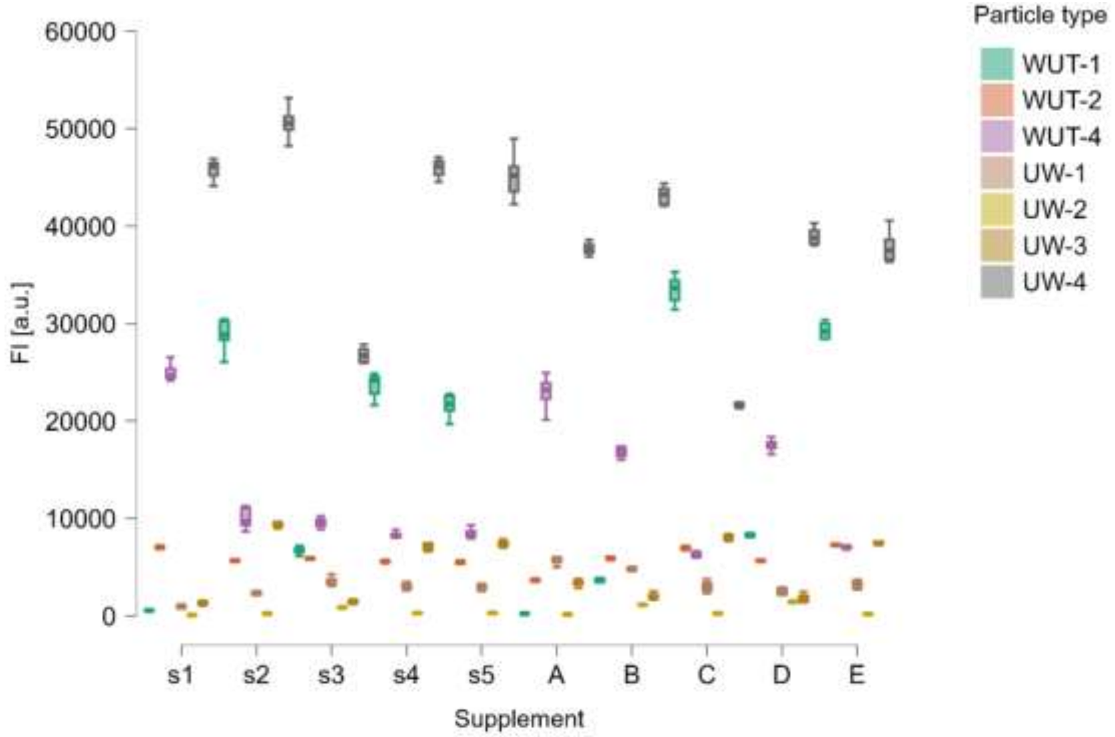

Figure S2. Classification accuracy of PLS-DA models calculated for three supplement sets (SET 1, SET 2 and combined SET 3, Table S2) based on full spectra: UV–Vis absorbance (A, B) and fluorescence intensity (C, D) data for the test sets, obtained using eight types of particles (Table S1). Columns represent mean ± SD; n=3.

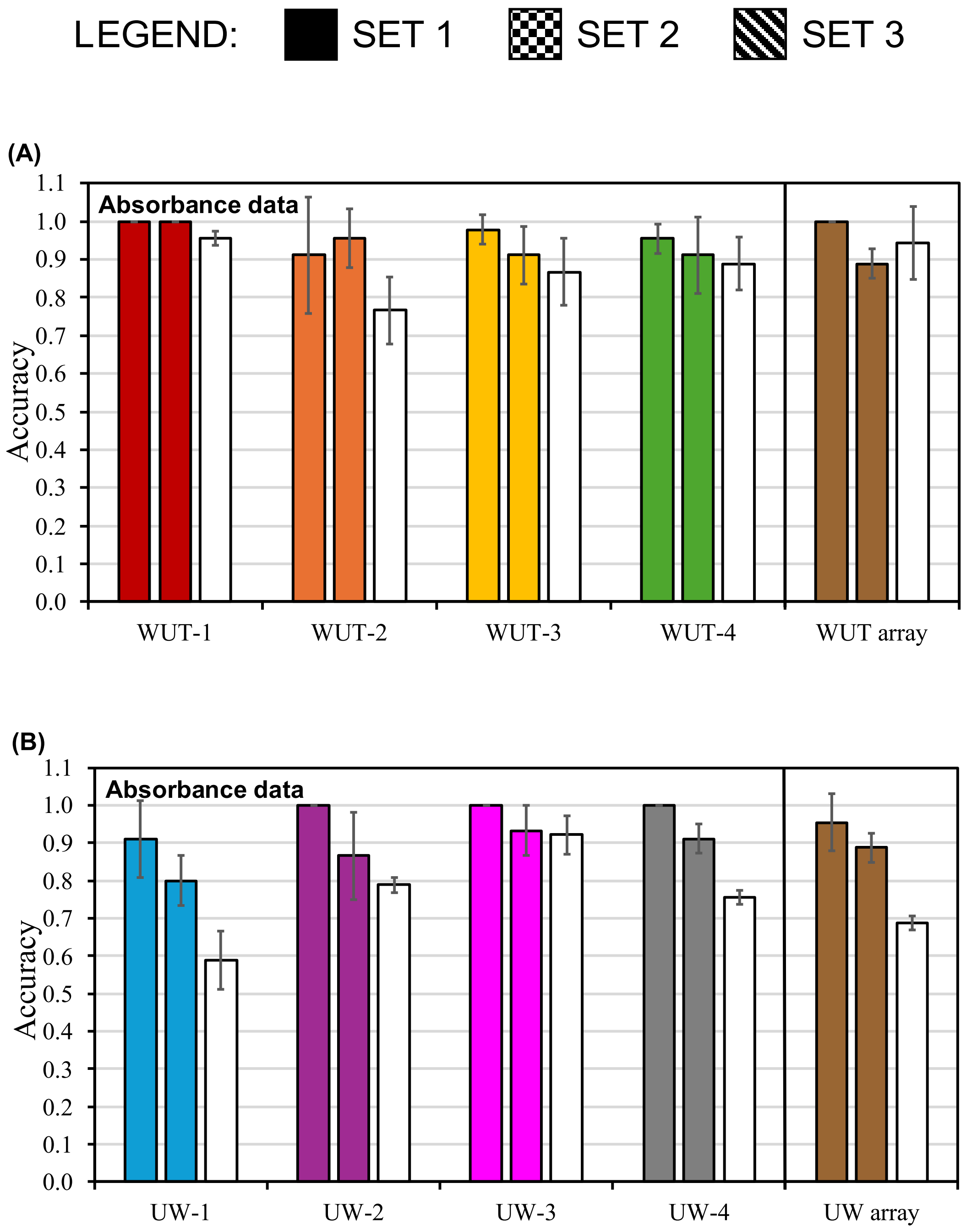

(C)

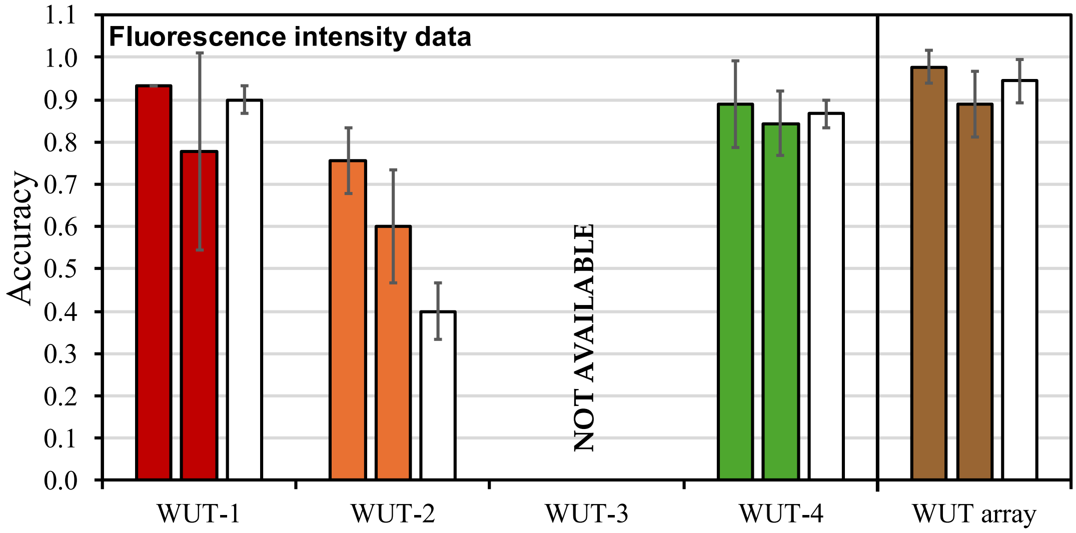

Fluorescence intensity data
Accuracy
1.1
1.0
0.9
0.8
0.7
0.6
0.5
0.4
0.3
0.2
0.1
0.0
NOT AVAILABLE
WUT-1
WUT-2
WUT-3
WUT-4
WUT array

(D)

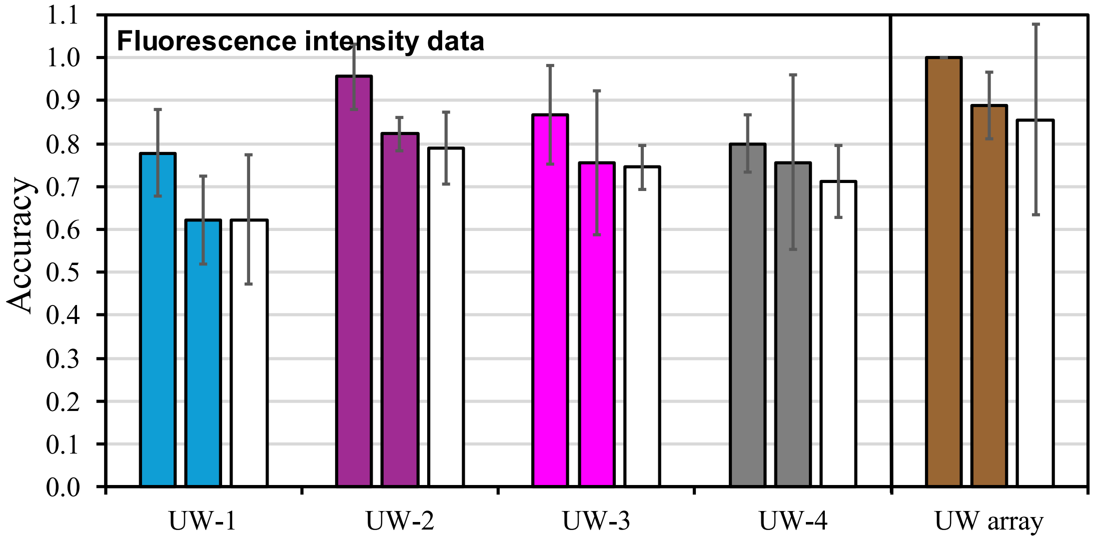

Fluorescence intensity data
Accuracy
1.1
1.0
0.9
0.8
0.7
0.6
0.5
0.4
0.3
0.2
0.1
0.0
UW-1
UW-2
UW-3
UW-4
UW array

Figure S3. Comparison of the accuracy of KNN classification models in the analysis of SET 3 dietary supplements based on UV–Vis absorbance (A, B) and fluorescence intensity (C, D) data. Columns represent mean ± SD; n=3.

(A)

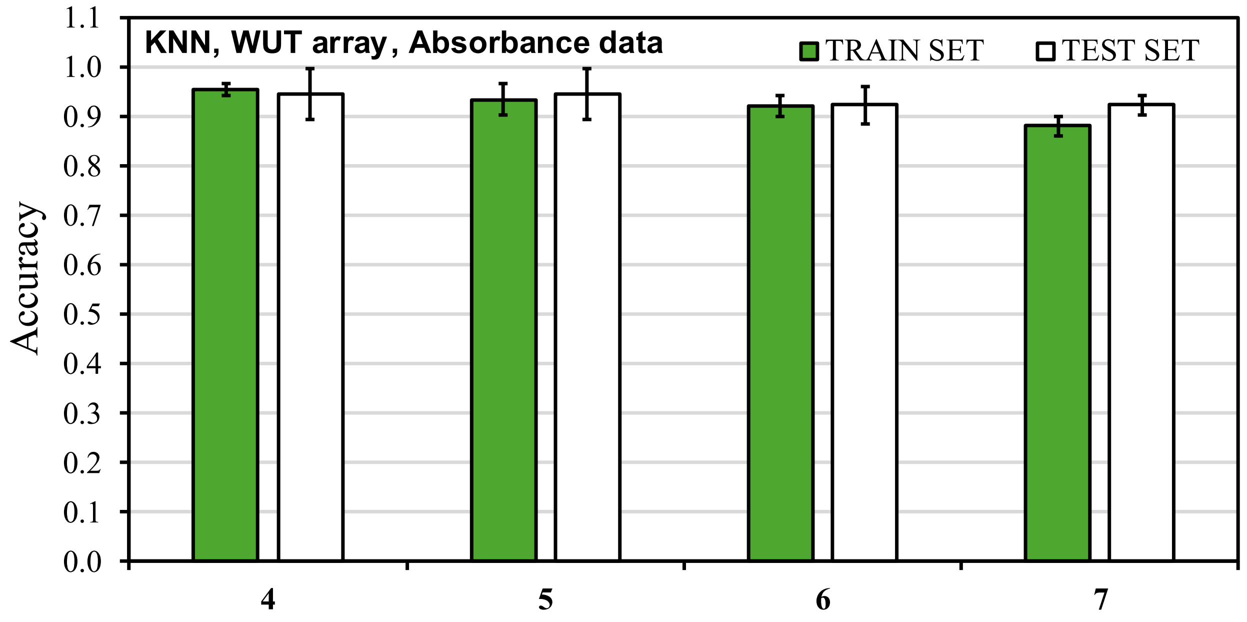

(B)

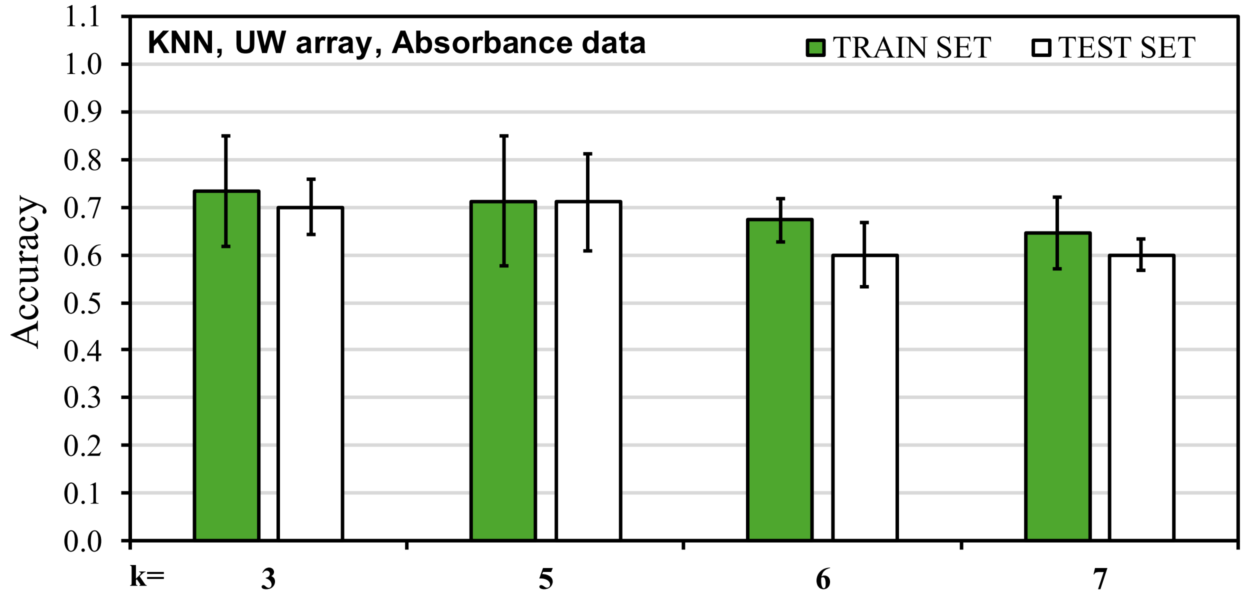

(C)

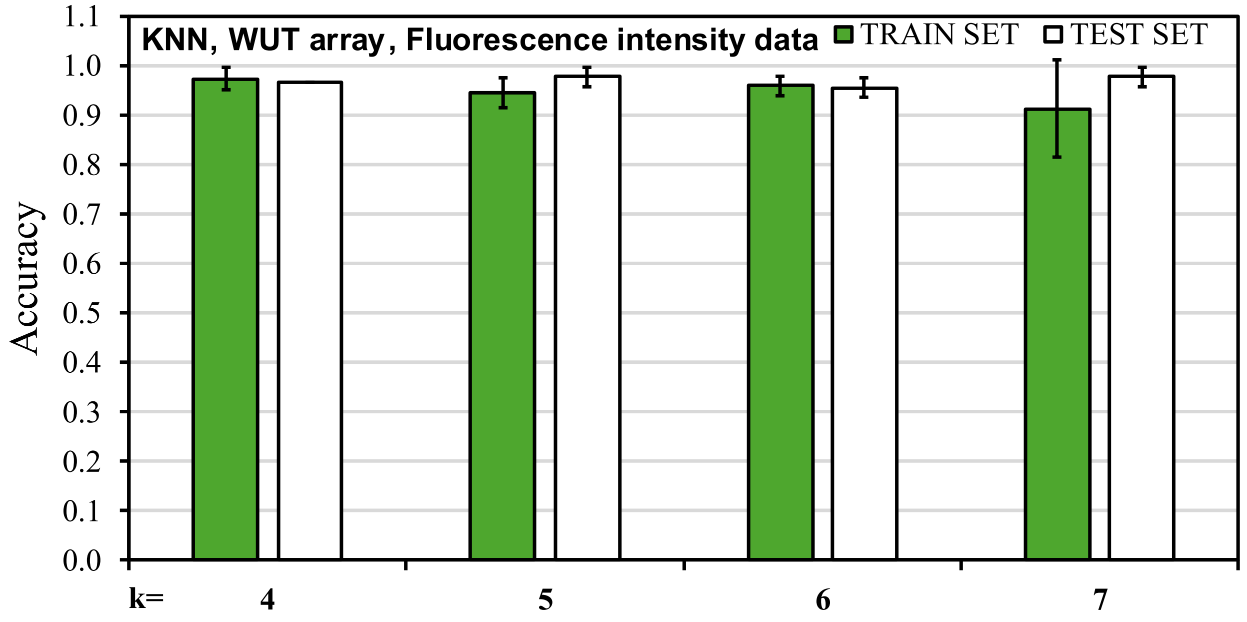

KNN, WUT array, Fluorescence intensity data
TRAIN SET
TEST SET
Accuracy
1.1
1.0
0.9
0.8
0.7
0.6
0.5
0.4
0.3
0.2
0.1
0.0
k=
4
5
6
7

(D)

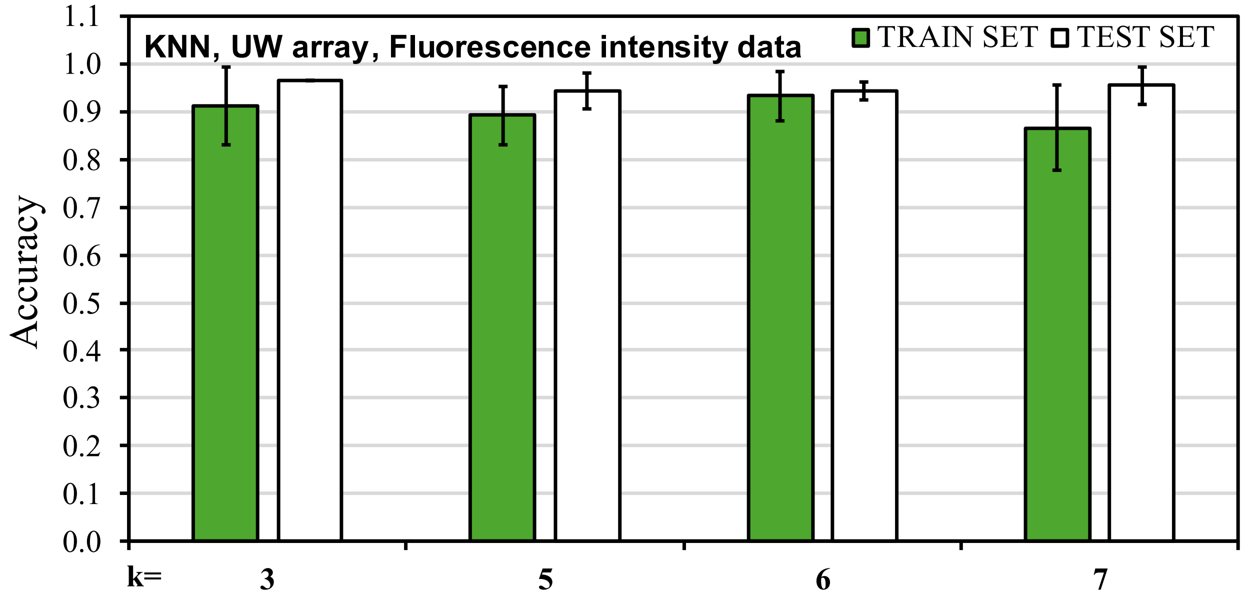

KNN, UW array, Fluorescence intensity data
TRAIN SET
TEST SET
Accuracy
1.1
1.0
0.9
0.8
0.7
0.6
0.5
0.4
0.3
0.2
0.1
0.0
k=
3
5
6
7
